# A cross-tissue transcriptome-wide association study identifies novel candidate genes associated with brain glymphatic system function

**DOI:** 10.1186/s13041-025-01258-y

**Published:** 2025-12-05

**Authors:** Xiaoyang Zhu, Shengjie Wang, Shuaiqi Zhang, Zhiyuan Liu, Na Wang, Shu Wang, Nixia Yang

**Affiliations:** 1 Binzhou Medical University, Yantai, Shandong China; 2 Qingdao Traditional Chinese Medicine Hospital, Qingdao Hiser Hospital Affiliated of Qingdao University, No. 4 Renmin Road, Shibei District, Qingdao City, Shandong Province China

**Keywords:** Glymphatic system, Cross-tissue TWAS, UTMOST, Colocalization, Mendelian randomization, DTI-ALPS

## Abstract

The glymphatic system plays a key role in brain waste clearance, but its genetic regulation remains poorly understood. Diffusion Tensor Image Analysis along the Perivascular Space (DTI-ALPS) index is a non-invasive imaging biomarker to asses glymphatic system activity. We integrated mean DTI-ALPS genome-wide association study (GWAS) data from 31,021 individuals of European ancestry with GTEx v8 multi-tissue eQTL data to perform transcriptome-wide association studies (TWAS) using Unified Test for Molecular Signature (UTMOST) and Functional Summary-based Imputation (FUSION). Gene-level associations were further validated by Multi-marker Analysis of Genomic Annotation (MAGMA). Causal inference was conducted using *cis*-Mendelian randomization (*cis*-MR) and summary-data-based Mendelian randomization (SMR), while colocalization was applied to provide evidence of strong associations between two traits within a single genetic region, thereby ensuring the stability of the MR conclusions. TWAS identified 17 candidate genes (AGBL5-IT1, CENPA, CGREF1, DNAJC5G, EMILIN1, GCAT, KHK, MAPRE3, OTOF, PLCL1, PREB, RBM43, RFTN2, SERPIND1, SNAP29, TRIOBP, and UCN), among which six protein-coding genes (TRIOBP, MAPRE3, EMILIN1, KHK, GCAT, and CGREF1) were further validated by MAGMA. *Cis*-MR provided evidence for the causal effects of these six genes, while colocalization supported that the MR conclusions were stable for four of them (TRIOBP, MAPRE3, EMILIN1, and GCAT). Finally, SMR identified three genes (TRIOBP, GCAT, and MAPRE3) that showed consistent and robust associations with DTI-ALPS across multiple tissues. These findings provide statistical evidence for genetic regulation of glymphatic function.

**Supplementary Information:**

The online version contains supplementary material available at 10.1186/s13041-025-01258-y.

## Introduction

The glymphatic system (GS), a recently discovered lymphatic-like clearance pathway in the central nervous system, facilitates the dynamic circulation of cerebrospinal fluid and interstitial fluid through perivascular spaces. It plays a crucial role in the removal of metabolic waste (such as β-amyloid), the regulation of neuroinflammation, and the maintenance of homeostasis [1]. GS dysfunction operates both upstream in neurodegeneration—including Alzheimer’s disease—via reduced clearance of β-amyloid and tau [2], and downstream of cerebrovascular pathology and white-matter injury [3, 4] a bidirectional architecture consistent with a feed-forward loop in which vascular and white-matter alterations blunt clearance and impaired clearance accelerates protein aggregation and tissue stress [5].

In recent years, the Diffusion Tensor Image Analysis along the Perivascular Space (DTI-ALPS) index, derived from diffusion tensor imaging, has been proposed as a non-invasive imaging biomarker to assess glymphatic activity. It quantifies the anisotropy of water diffusion in perivascular spaces, serving as a valuable tool for investigating glymphatic function in the living human brain. A higher DTI-ALPS index indicates better directional organization of white matter tracts and greater local structural coherence, reflecting a lower degree of glymphatic dysfunction [6]. Current research predominantly focuses on the clinical phenotypic correlations of this index, while the underlying gene regulatory network remains largely unexplored [7].

In this context, a large-scale genome-wide association study (GWAS) involving 31,021 individuals from the UK Biobank revealed the genetic complexity of the ALPS index, identifying 17 genome-wide significant loci mapped to 161 candidate genes [8]. Although this progress provides valuable insights into the multidimensional regulation of the glymphatic system, most of the variants identified by GWAS are located in non-coding regions, posing challenges for functional interpretation. Furthermore, the complex linkage disequilibrium (LD) structure limits the precise identification of causal genes [9, 10].

Transcriptome-wide association studies (TWAS), which integrate expression quantitative trait loci (eQTL) data with GWAS findings, provide an effective approach to linking non-coding variants with gene expression regulation. This method has successfully identified pathogenic genes in neurodegenerative diseases such as Alzheimer’s disease [11]. Cross-tissue TWAS approaches, incorporate eQTL information from multiple tissues, capturing both shared regulatory signals and tissue-specific effects [12]. This strategy is particularly suitable for studying the glymphatic system, as its functions involve multiple brain regions and may be influenced by peripheral immune regulation [13]. Previous cross-tissue analyses have revealed key genes in multisystem disorders such as migraine [14] and autism spectrum disorder [15], highlighting the unique value of this approach in elucidating neurovascular-immune interaction networks.

This study, based on recently published genome-wide association data for the DTI-ALPS index, integrated multi-tissue eQTL data from the Genotype-Tissue Expression (GTEx) project v8 and, for the first time, applied the Unified Test for Molecular Signature (UTMOST) to conduct a cross-tissue TWAS analysis [8]. Gene–phenotype associations were evaluated at the single-tissue level using Functional Summary-based Imputation (FUSION) [16], UTMOST emphasizes coordinated genetic effects across tissues and improves detection of susceptibility genes for complex phenotypes such as glymphatic function, whereas FUSION focuses on tissue-specific regulatory mechanisms and represents one of the most widely used single-tissue TWAS frameworks. The two approaches are complementary, and we prioritized the intersection of their signals as the primary result set to enhance robustness and reduce false positives. And further replication was conducted with Multi-marker Analysis of Genomic Annotation (MAGMA) [17]. Mendelian randomization (MR) uses genetic variants as instrumental variables to infer causal effects between exposures and outcomes [18]. *Cis*-MR restricts instruments to variants located near the gene encoding the exposure to reduce pleiotropic bias, while summary-data-based MR (SMR) integrates GWAS and eQTL summary data to link gene expression with complex traits [19, 20]. Colocalization is used to evaluate whether two traits share the same genetic association signal, and when focusing on variants within a single locus, a positive colocalization finding typically implies a non-zero MR estimate [21, 22]. For the candidate genes identified above, causal inference was performed using *cis*-MR and SMR, while Bayesian colocalization was applied to assess whether the associations at each locus were driven by shared causal variants, thereby ensuring the stability of MR conclusions.

## Methods

### Study design

**Fig. 1:**
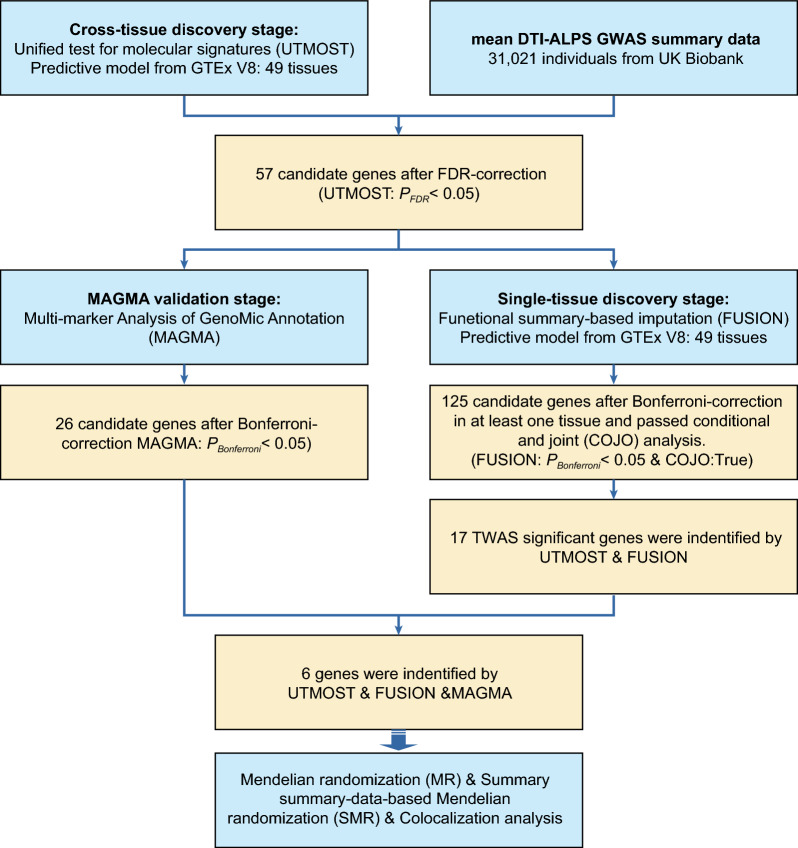
The flowchart of this study. GWAS, Genome-wide association study; GTEx, Genotype-Tissue Expression Project; TWAS, Transcriptome-wide association study; UTMOST, Unified test for molecular signatures; FUSION, Functional summary-based imputation; MAGMA, Multi-marker analysis of genomic annotation; MR, Mendelian randomization; COJO, Conditional and joint analysis; DTI-ALPS, Diffusion tensor imaging–analysis along the perivascular space; SMR, Summary-data-based Mendelian randomization

The analysis process is illustrated in Fig. 1.

### Data sources

The genome-wide association data for the average DTI-ALPS index were derived from a GWAS involving 31,021 individuals of white European ancestry from the UK Biobank. Participants were restricted to individuals of white descent to minimize the effects of population stratification. The ALPS index was calculated based on diffusion tensor imaging by placing regions of interest in bilateral projection and association fiber areas. Diffusivities along the x-, y-, and z-axes were extracted and averaged bilaterally to compute the mean DTI-ALPS index. To mitigate confounding from white-matter morphology and to more closely index glymphatic function, the original study further included global white-matter integrity measures, including mean fractional anisotropy, mean diffusivity, and other related indices, as covariates in the GWAS models [8].

The eQTL data were obtained from the GTEx project v8, which includes gene expression data from 838 postmortem donors (https://ftp.ebi.ac.uk/pub/databases/spot/eQTL/imported/GTEx_V8). The sample sizes varied across tissues, ranging from 73 samples in the renal cortex to 706 samples in skeletal muscle [23, 24].

### UTMOST

Cross-tissue TWAS was performed with UTMOST [12] using expression prediction models trained on GTEx v8, whose reference population is predominantly of European ancestry, consistent with the ancestry of the DTI-ALPS GWAS cohort used for the outcome. Compared to single-tissue approaches, UTMOST provides higher imputation accuracy and enables the construction of valid imputation models for a greater number of genes. Subsequently, the Generalized Berk–Jones (GBJ) test was applied to aggregate gene–trait associations by leveraging the covariance structure of single-tissue statistics. After applying false discovery rate (FDR) correction, an FDR threshold of < 0.05 was considered statistically significant [25].

### FUSION analysis

FUSION (http://gusevlab.org/projects/fusion/) was used to integrate disease-specific GWAS results with eQTL data from 49 tissues in the GTEx v8 project, aiming to identify potential gene–trait associations [16]. The analysis pipeline first utilized European samples from the 1000G as a reference to estimate the LD structure between single nucleotide polymorphisms (SNPs) and gene expression prediction models. Multiple prediction algorithms—including BLUP, BSLMM, LASSO, Elastic Net, and the top1 model—were applied to model the regulatory effects of SNPs on gene expression. The model with the best performance in cross-validation was selected to generate gene expression weights.

These gene weights were then integrated with GWAS Z-scores for the target trait to perform the TWAS. To identify independent genetic signals, conditional and joint (COJO) analysis was conducted within the FUSION framework [26]. By accounting for LD among markers, this analysis provides a more comprehensive understanding of the genetic architecture [27]. Genes that remained significant after conditioning were defined as jointly significant, whereas those that lost significance were considered non-contributory.

To determine statistical significance, candidate genes were required to meet the following criteria: (1) FDR < 0.05 in the cross-tissue analysis, and (2) significance in at least one tissue-specific analysis after Bonferroni correction (p < 0.05) and confirmation via COJO analysis.

### MAGMA analysis

Gene-level association was performed with MAGMA (v1.08) to identify trait-related genes, the software captures signals arising from multiple low-effect SNPs, default settings aggregate SNP-level summary statistics into gene scores, quantifying gene–phenotype associations [17].

### *Cis*-MR and colocalization analysis

We first selected *cis*-acting SNPs strongly associated with tissue-specific gene expression traits using a *p*-value threshold of 5 × 10^−8^ within a 1000 kb *cis*-region. To increase the number of testable genes, we re-screened for strongly associated *cis*-SNPs in cases where no SNPs were identified under the strict threshold, using a relaxed threshold of 1 × 10^−5^ within the same 1000 kb window. SNPs obtained under both thresholds were clumped using the European reference panel from the 1000 Genomes Project based on an LD threshold of r^2^ = 0.001. After harmonization with outcome data, we conducted Mendelian randomization using the Wald ratio method. Results with *p*-values below the Bonferroni-corrected threshold of 0.0012 (0.05/42) were considered statistically significant. Subsequently, we queried the Ensembl database for the SNPs used in the MR analyses; where potential confounding or horizontal pleiotropy was indicated, we interpreted the exposure–outcome relationship as an association rather than causation.

Bayesian colocalization analysis was performed on 66 gene–trait pairs identified in previous analyses to evaluate whether the GWAS and eQTL signals colocalize at a shared causal variant. We considered a posterior probability (PP.H4) > 0.8 as strong evidence of a shared causal variant between the GWAS and eQTL signals. Gene–trait pairs that passed both the *cis*-MR and colocalization criteria were considered to have putative causal relationships.

### SMR analysis

To further infer causality, we applied the SMR method to tissue-specific gene expression traits that showed causal associations in both the *cis*-MR and colocalization analyses [19]. This method is designed to assess causal relationships between molecular traits and complex phenotypes. The analysis was restricted to *cis*-eQTLs, using significance thresholds of p < 5 × 10^−8^ and a minor allele frequency > 1% for eQTL selection. SMR results with *p*-values below the Bonferroni-corrected threshold of 0.002941 (0.05/17) were considered robust evidence of a causal relationship, and these associations were further supported by passing the HEIDI test (p > 0.1).

## Results

### Cross-tissue and single-tissue TWAS results

In the cross-tissue TWAS analysis, a total of 282 susceptibility genes were identified with *p*-values < 0.05, among which 57 genes remained significant after FDR correction. In the single-tissue TWAS analysis, 125 genes were found to be significant (Bonferroni-corrected p < 0.05) in at least one tissue and were further validated through COJO analysis. Seventeen genes overlapped between the cross-tissue and single-tissue analyses, of which 94.12% were protein-coding genes.

**Fig. 2:**
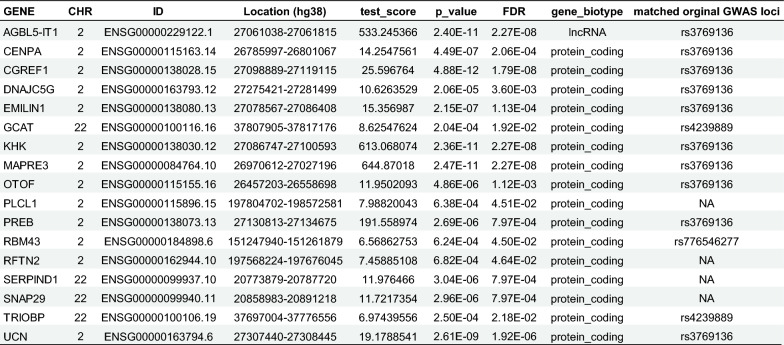
Important genes associated with the brain lymphatic system identified by cross-tissue UTMOST and FUSION analysis. This figure presents genes significantly associated with brain glymphatic system across tissues, as identified using UTMOST and FUSION transcriptome-wide association analyses

Figure 2 presents the cross-tissue TWAS results of the 17 genes identified in both analyses. The full list of significant genes from the cross-tissue TWAS is provided in Table S1, and all results from the single-tissue TWAS are listed in Table S2.

### MAGMA analysis

Based on the MAGMA gene-level analysis, 26 genes were identified as significantly associated with the DTI-ALPS phenotype (Bonferroni-corrected p < 0.05).

To ensure the robustness of the findings, we intersected the gene sets obtained from the cross-tissue TWAS, single-tissue TWAS, and MAGMA analyses, resulting in six genes that were consistently significant across all three methods. These genes—TRIOBP, KHK, MAPRE3, GCAT, EMILIN1, and CGREF1—are all protein-coding.

**Fig. 3:**
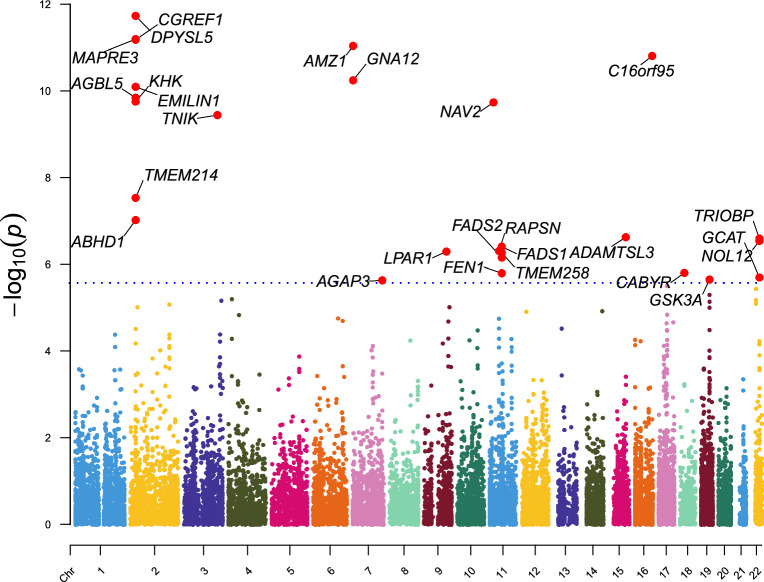
This figure illustrates the gene-level association results derived from the MAGMA analysis. The y-axis represents the − log_10_(p) values, indicating the statistical significance of association, with higher values reflecting stronger evidence. The x-axis denotes the chromosome number (CHR 1 to 22). Red dots highlight genes that surpass the Bonferroni-corrected significance threshold, while the blue dashed line marks this threshold. Only genes above this line are considered statistically significant after correction for multiple testing

**Fig. 4:**
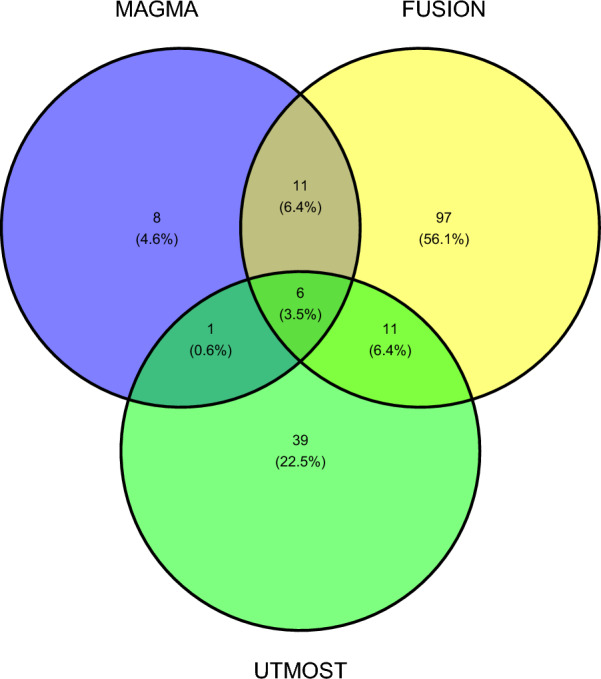
This Venn diagram illustrates the overlap of key genes associated with glymphatic system function identified by three transcriptome-wide association methods: MAGMA, FUSION, and UTMOST. Specifically, MAGMA identified 26 significant genes, FUSION identified 125, and UTMOST (via cross-tissue analysis) identified 57 genes. A total of 6 genes were shared across all three methods

In the FUSION analysis, these genes showed significant associations across 44 tissues, with a total of 66 gene–tissue pairs. MAGMA results are presented in Fig. 3 and Table S3. The overlap of significant genes across UTMOST, FUSION, and MAGMA is illustrated in Fig. 4.

### *Cis*-Mendelian randomization and colocalization

**Fig. 5:**
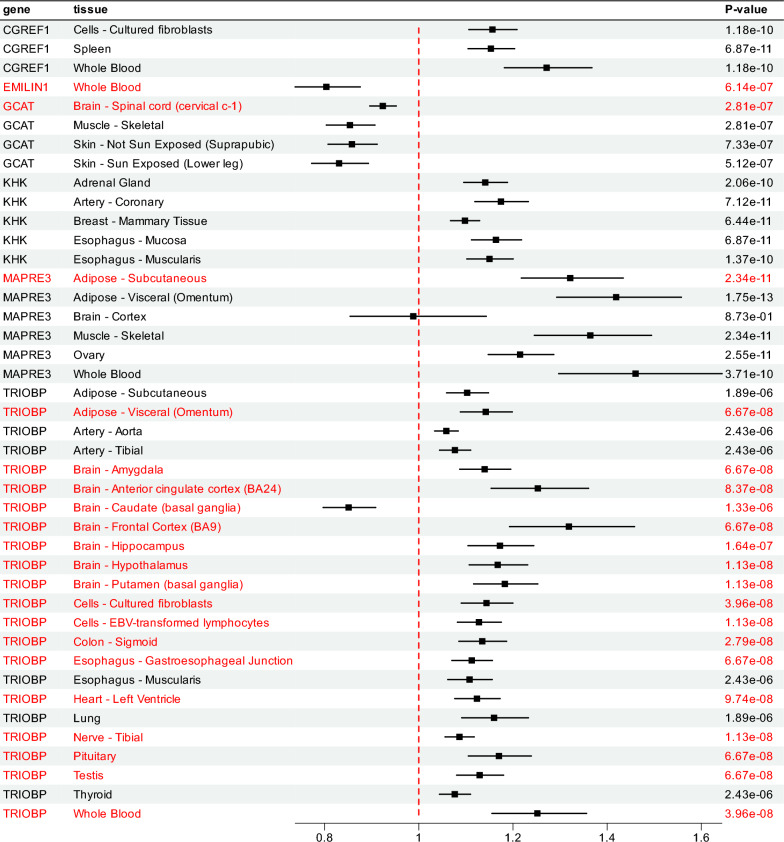
MR analysis results of causal effects between genes and DTI-ALPS. Genes with strong colocalization evidence with the DTI-ALPS phenotype (PPH4 > 0.8) are highlighted in red

Using a stringent *p*-value threshold of 5 × 10^−8^, *cis*-Mendelian randomization identified five genes (TRIOBP, MAPRE3, KHK, GCAT, and CGREF1) across 28 tissues, corresponding to 32 gene expression traits with 32 instrumental variables. Under the relaxed threshold of 1 × 10^−5^, four additional genes (EMILIN1, CGREF1, TRIOBP, and MAPRE3) were identified across 8 tissues, yielding 10 additional gene expression traits and their associated instrumental variables. The Wald ratio method revealed that, except for MAPRE3 in the Brain-Cortex tissue, all results remained significant after Bonferroni correction. Among the final 41 positive results, nine may be influenced by confounding; the genes with confirmed causal evidence are TRIOBP, MAPRE3, KHK, EMILIN1, GCAT, and CGREF1. Detailed results from the *cis*-Mendelian randomization analysis are presented in Fig. 5 and Table S4.

Among the 66 gene–tissue pairs encompassing six genes across 44 tissues, four genes (TRIOBP, MAPRE3, EMILIN1, and GCAT) demonstrated strong colocalization in 20 expression traits across 19 tissues (PP.H4 > 0.8), supporting shared causal variants between eQTL and GWAS signals and reinforcing the non-zero causal estimates obtained by Mendelian randomization. Gene–tissue pairs with PP.H4 ≤ 0.8 were considered indicative rather than confirmatory, and were not prioritized as potential targets. Comprehensive colocalization results are summarized in Table S5 and visualized in Fig. S1. The distribution of tissues corresponding to the previously identified single-tissue positive results is presented in Supplementary Fig. S2.

### SMR-validated genes

A total of three genes—GCAT, TRIOBP, and MAPRE3—had valid instrumental variables for gene expression traits across 17 distinct tissues. All SMR results remained statistically significant after Bonferroni correction and passed the HEIDI test. Specifically, elevated expression of TRIOBP was significantly associated with increased DTI-ALPS values in 15 tissues, including multiple brain regions. GCAT expression in the Brain_Spinal_cord_cervical_c-1 tissue was significantly associated with decreased DTI-ALPS, while MAPRE3 expression in Adipose-Subcutaneous was positively associated with DTI-ALPS.

**Fig. 6:**
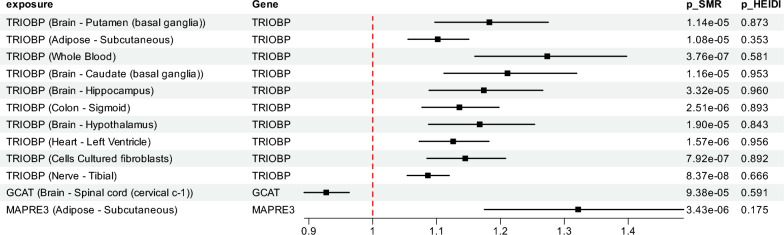
SMR results of causal effects between genes and DTI-ALPS

Notably, although EMILIN1 expression in blood had previously shown strong evidence of causality via colocalization and *cis*-MR, it could not be tested in the SMR analysis due to the lack of sufficient instrumental variables. SMR results are provided in Table S6 and Fig. 6.

## Discussion

In this study, we identified six protein-coding genes—TRIOBP, KHK, MAPRE3, GCAT, EMILIN1, and CGREF1—that are potentially involved in glymphatic system function, through the integration of cross-tissue and single-tissue TWAS methods with MAGMA analysis. Subsequent causal inference using *cis*-Mendelian randomization, colocalization analysis, and SMR provided converging evidence for three of these genes (TRIOBP, GCAT, and MAPRE3) exerting tissue-specific causal effects on glymphatic activity.

First, TWAS results showed that elevated expression of TRIOBP in 28 tissues was significantly associated with increased DTI-ALPS values, suggesting that TRIOBP may play a role in glymphatic clearance or glial cell activity. Previous studies have demonstrated that TRIOBP is essential for cytoskeletal stability, particularly through its regulation of F-actin polymerization and microtubule organization [28]. Analysis of Human Protein Atlas single-cell datasets showed significant enrichment of TRIOBP in astrocytes and endothelial cells across major CNS regions, including the cortex, hippocampus, amygdala, basal ganglia, thalamus, hypothalamus, midbrain, cerebellum, brainstem, and spinal cord. Consistent with these patterns, TWAS showed robust associations between genetically predicted TRIOBP expression and the DTI-ALPS index in amygdala, cortex (BA9 and anterior cingulate BA24), basal ganglia (caudate and putamen), hippocampus, and hypothalamus, suggesting contributions of astroglial and endothelial compartments to glymphatic alterations [29]. Given that DTI-ALPS reflects the permeability and waste clearance efficiency of the brain’s glymphatic system [30], upregulation of TRIOBP may enhance cytoskeletal stability in astrocytes or microglia, thereby facilitating the structural integrity or function of brain fluid clearance pathways. Moreover, downregulation of TRIOBP in Sertoli cells has been shown to disrupt intercellular junctions. Since the glymphatic system relies on tight connections between astrocytes, TRIOBP may indirectly modulate intercellular diffusion of interstitial fluid by regulating cell–cell junctions, thereby enhancing glymphatic clearance capacity [31]. Similarly, TRIOBP mutations have been linked to ultrastructural defects in the electron-dense zone of ciliary rootlets, which are associated with extracellular matrix stability. Because glymphatic function depends on the integrity of the perivascular basement membrane, TRIOBP may enhance water conductance in perivascular spaces by stabilizing the extracellular matrix [32]. Additionally, the TRIOBP-1 isoform can regulate actin dynamics at synaptic terminals via its pleckstrin homology and coiled-coil domains, potentially modulating neuronal electrical activity and indirectly supporting glymphatic function [33].

Second, in Brain_Spinal_cord_cervical_c-1, Mendelian randomization suggested a negative association between GCAT expression and the DTI-ALPS index; this may relate to cervico-spinal glymphatic pathways. CSF–ISF exchange proceeds along perivascular astroglial networks throughout the neuraxis, and outflow to cervical lymph nodes provides a continuous route for extracranial clearance; GCAT may contribute by modulating astroglial metabolic–osmotic balance at perivascular interfaces, consistent with its signal in Brain_Spinal_cord_cervical_c-1 [34]. On the other hand, GCAT plays a key role in mitochondrial glycine metabolism and the one-carbon metabolic pathway, regulating cellular energy homeostasis and redox balance [35]. Previous studies have shown that metabolic dysfunction can compromise white matter structural integrity, thereby disrupting interstitial fluid dynamics in the brain [36, 37]. Given that DTI-ALPS is sensitive to such structural changes, aberrant expression of GCAT may impair glymphatic clearance through metabolic disruption. Moreover, GCAT protein levels are markedly reduced in the brains of patients with late-onset Alzheimer’s disease, leading to the accumulation of its downstream metabolite methylglyoxal and its glycation end-products. These end-products are known neurotoxins that can induce glial cell activation and disrupt aquaporin-4 polarity, ultimately impairing glymphatic clearance [38]. Taken together, these findings suggest that dysregulated GCAT expression may impair brain fluid clearance via metabolic imbalance, accumulation of neurotoxic metabolites, and disruption of aquaporin-4 polarity.

Finally, we found that the expression level of MAPRE3 was also causally associated with the DTI-ALPS index. As a key member of the microtubule-associated protein family, MAPRE3 primarily regulates microtubule dynamic stability and cytoskeletal architecture, thereby influencing intracellular material transport [39]. The flow of brain interstitial fluid critically depends on astrocyte polarity and the structural support of the microtubule network. Thus, MAPRE3 may influence the routing and efficiency of fluid movement in the brain by modulating microtubule stability [40, 41]. We observed that increased expression of MAPRE3 was associated with higher DTI-ALPS values, suggesting a potential role in maintaining the structural integrity of cerebral clearance pathways. In addition, previous studies have shown that MAPRE3 can cooperate with adhesion G protein-coupled receptor F5 to regulate extracellular matrix remodeling. While the receptor alters the structure of perivascular tissue by modulating cell–matrix adhesion signaling, MAPRE3 may participate in the mechanical regulation of the extracellular environment, thereby enhancing fluid transport efficiency through perivascular spaces [42]. Furthermore, glymphatic clearance has been shown to be significantly enhanced during sleep. MAPRE3 may contribute to this process through its interaction with the activity-dependent neuroprotective protein–SIRT1 complex, which is involved in circadian signaling pathways, thereby modulating the sleep–wake cycle and enhancing nighttime brain clearance [43]. MAPRE3 and GCAT, as key genes involved in microtubule assembly, show causal effects on GS function. Microtubule dynamics represent a critical pathogenic mechanism in tau-related neurodegenerative disorders driven by the Microtubule-Associated Protein Tau gene [44]. This mechanistic link suggests that impaired microtubule formation may underlie the previously observed associations between DTI-ALPS and multiple Microtubule-Associated Protein Tau-related neurological diseases, including frontotemporal dementia [45], Parkinson’s disease [46], and Alzheimer’s disease [6].

In addition, although EMILIN1, CGREF1, and KHK did not pass all downstream causal inference analyses, the expression of EMILIN1 in blood showed evidence of a causal relationship with DTI-ALPS through *cis*-Mendelian randomization and colocalization. However, due to the lack of sufficient instrumental variables, causal inference using SMR could not be conducted. These results suggest that EMILIN1 may still represent a potential therapeutic target for improving glymphatic function.

Although CGREF1 and KHK were not causally linked to DTI-ALPS, they exhibited strong statistical associations with this trait and may serve as predictive indicators.

A key strength of this study lies in the application of the UTMOST framework, a multi-tissue transcriptome-wide association method that is more suitable for phenotypes such as DTI-ALPS, which are likely regulated by multiple tissues [47]. Compared with traditional genome-wide association approaches, transcriptome-wide association studies offer improved power for identifying protein-coding genes with therapeutic potential.

Lastly, due to ethical and financial constraints, randomized controlled trials cannot currently be used to investigate the genetic regulation of DTI-ALPS in human populations. This study therefore utilized transcriptome-based methods across multiple public cohorts to explore mechanistic pathways in an ethically and economically feasible manner.

In *cis*-MR, many genes lacked valid instrumental variables for causal inference. To address this, we relaxed the single nucleotide polymorphism significance threshold to 1 × 10^−5^. Nevertheless, a substantial number of gene–tissue pairs still lacked suitable instruments. Based on the recommendation of Stephen Burgess [22], we performed Bayesian colocalization analysis on the previously prioritized 66 gene–tissue traits to explore potential causal relationships with DTI-ALPS under the assumption of *cis*-regulatory effects. Notably, we identified 20 colocalized gene–trait pairs, all of which fell within the set of significant *cis*-Mendelian randomization results, underscoring the robustness of our findings. Of these, 14 provided evidence consistent with causality, whereas 6 indicated association without causal support. However, one exception was observed: in the *cis*-Mendelian randomization analysis, higher TRIOBP expression in the Brain-Anterior cingulate cortex (BA24) was associated with lower DTI-ALPS values, in contrast to its effects in other tissues. This inconsistency may be due to the limited robustness of *cis*-Mendelian randomization, which relies on a single variant for inference.

To address this limitation, we applied SMR, which allows for multiple instrumental variants and offers improved statistical stability. The subsequent analysis confirmed that higher TRIOBP expression in the Brain-Anterior cingulate cortex (BA24) was indeed associated with higher DTI-ALPS values, aligning with the broader expression pattern observed in other tissues.

Several methodological limitations should be acknowledged. First, TWAS analyses are prone to co-expression artifacts, often implicating false-positive genes located near true positive signals. To mitigate this, we performed COJO analysis after TWAS to reduce spurious associations; subsequent MR and colocalization analyses also help to attenuate such biases to some extent [48]. Second, MR has long been criticized for potential misuse. We applied it with caution, stringently filtering SNPs potentially confounded by pleiotropy, and incorporated colocalization as an auxiliary strategy to minimize erroneous inferences [49]. Third, the resolution of this study is restricted to the tissue level; due to the current scarcity of single-cell data and methodological limitations, higher cellular resolution could not yet be achieved. Fourth, the GWAS data for DTI-ALPS are available only in populations of European ancestry, and extrapolation to other ancestries should therefore be made with caution. Additionally, because of ethical and financial constraints, the findings have not been validated in randomized controlled trials, and further prospective cohort studies will be required to confirm the causal relationships proposed here.

## Supplementary Information

Supplementary Material 1.

Supplementary Material 2.

Supplementary Material 3.

Supplementary Material 4.

Supplementary Material 5.

Supplementary Material 6.

Supplementary Material 7.

Supplementary Material 8.

## Data Availability

This study analyzes publicly available datasets. eQTL data can be found at the GTEx v8 Project (https://ftp.ebi.ac.uk/pub/databases/spot/eQTL/imported/GTEx_V8); the GWAS data on mean DTI-ALPS originated from the GWAS Catalog (https://www.ebi.ac.uk/gwas/) under Accession Number GCST90454295.

## Abbreviations

GS: Glymphatic system
DTI-ALPS: Diffusion Tensor Image Analysis along the Perivascular Space
GWAS: Genome-wide association study
LD: Linkage disequilibrium
TWAS: Transcriptome-wide association studies
eQTL: Expression quantitative trait loci
UTMOST: Unified Test for Molecular Signature
FUSION: Functional Summary-based Imputation
MAGMA: Multi-marker Analysis of Genomic Annotation
MR: Mendelian randomization
SMR: Summary-data-based Mendelian randomization
COJO: Conditional and joint analysis
GBJ: Generalized Berk–Jones
FDR: False discovery rate
SNP: Single nucleotide polymorphism
