# Supplementary figures and images for: A cross-tissue transcriptome-wide association study identifies novel candidate genes associated with brain glymphatic system function
Source: Mol Brain. 2025 Dec 5;18:90. doi: 10.1186/s13041-025-01258-y (PMC12681178; doi:10.1186/s13041-025-01258-y)

**A**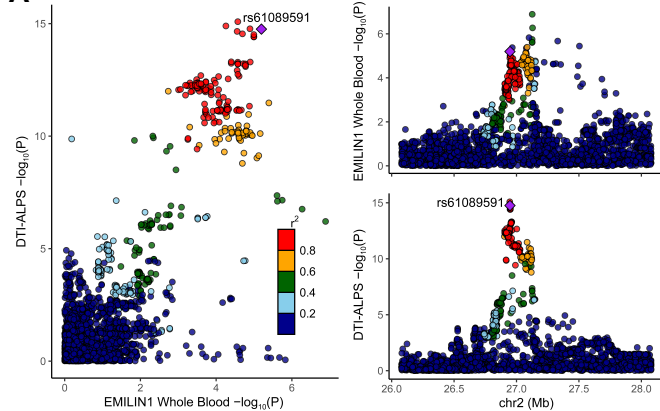**B**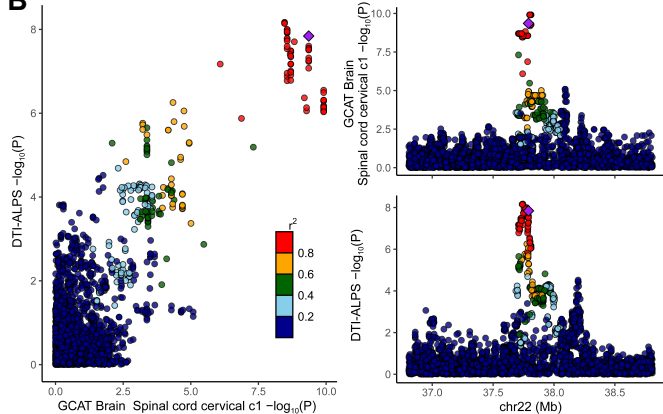**C**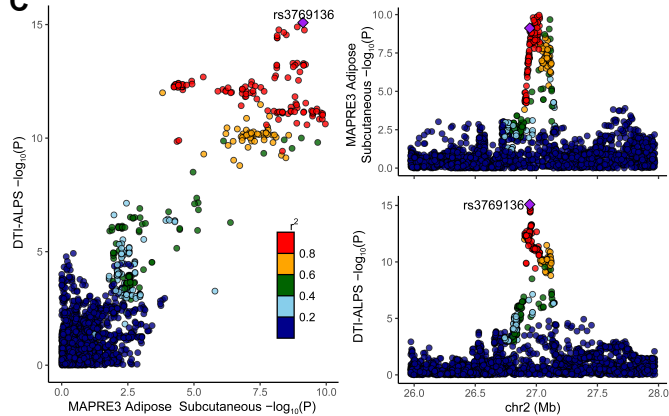**D**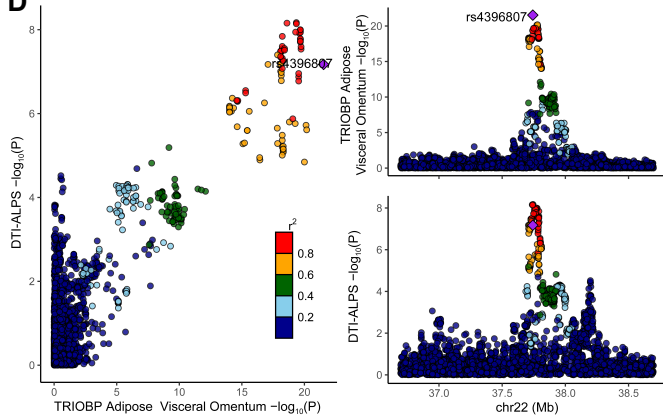

Supplement: Supplementary file 1 — Supplementary Material 1. [file 13041_2025_1258_MOESM1_ESM.pdf]

**E**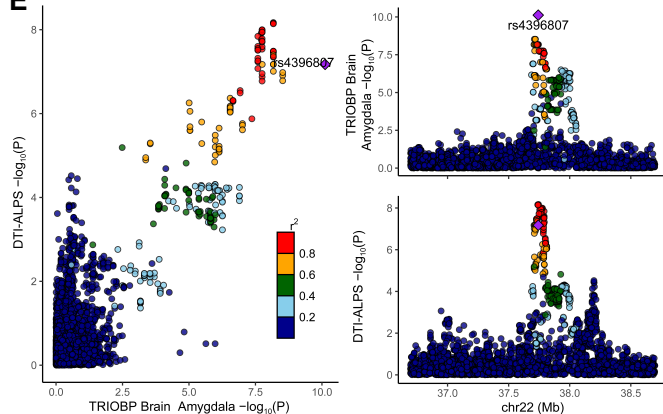**F**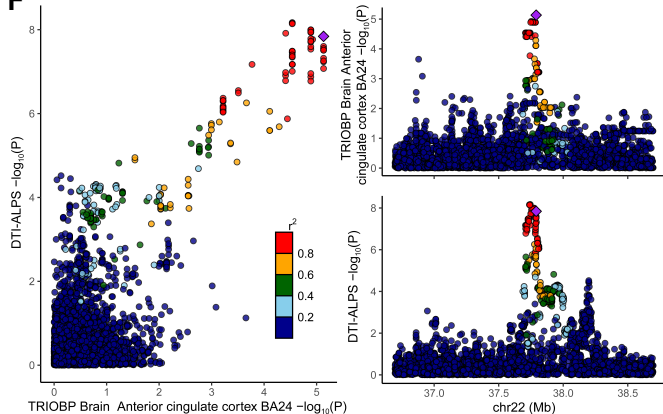**G**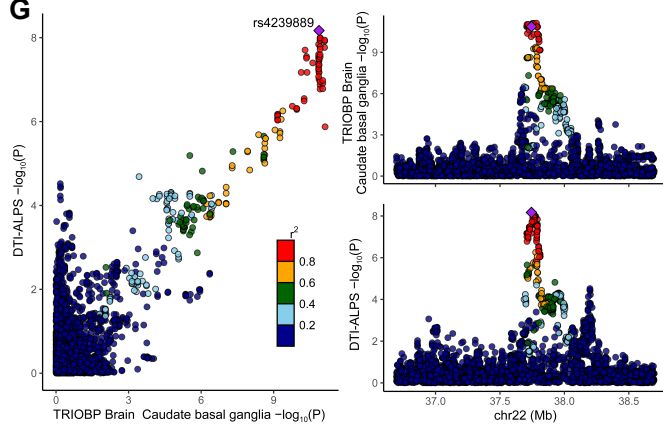**H**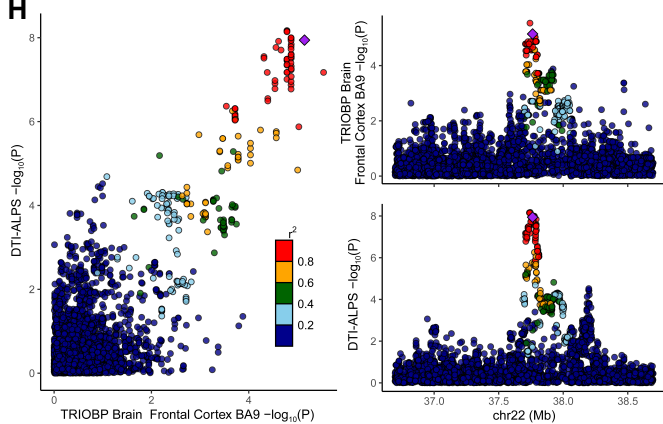

Supplement: Supplementary file 2 — Supplementary Material 2. [file 13041_2025_1258_MOESM2_ESM.pdf]

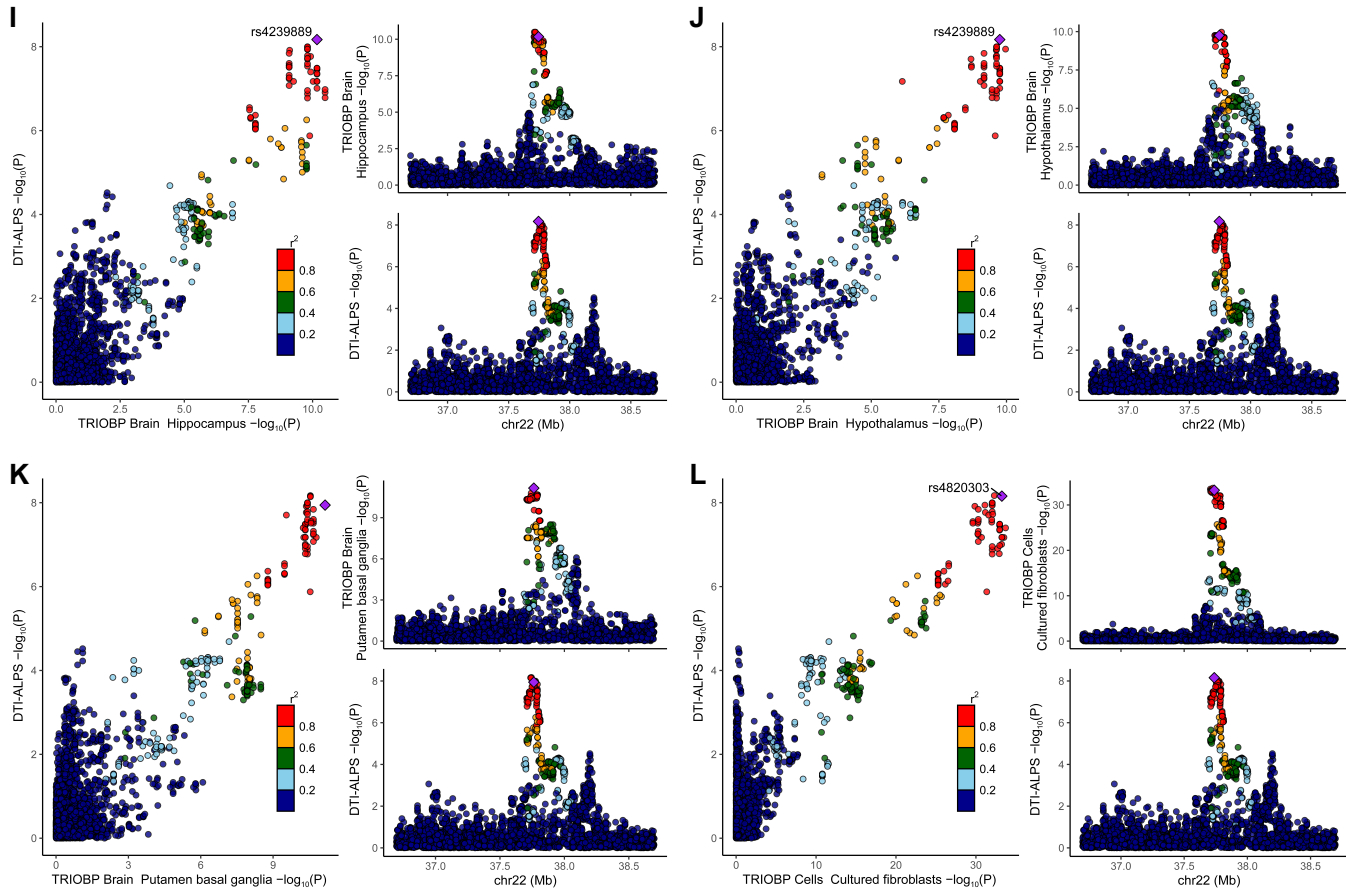

Supplement: Supplementary file 3 — Supplementary Material 3. [file 13041_2025_1258_MOESM3_ESM.pdf]

**M**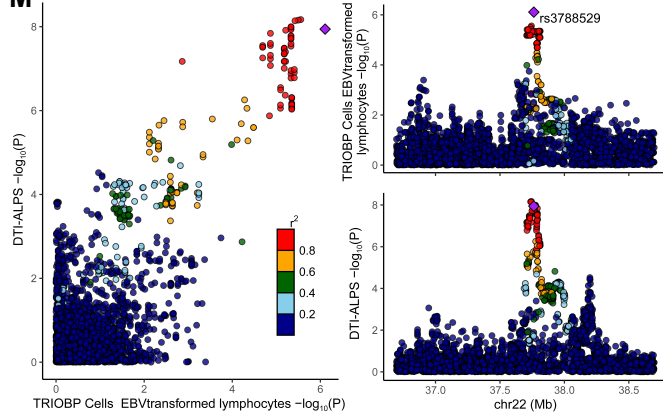**N**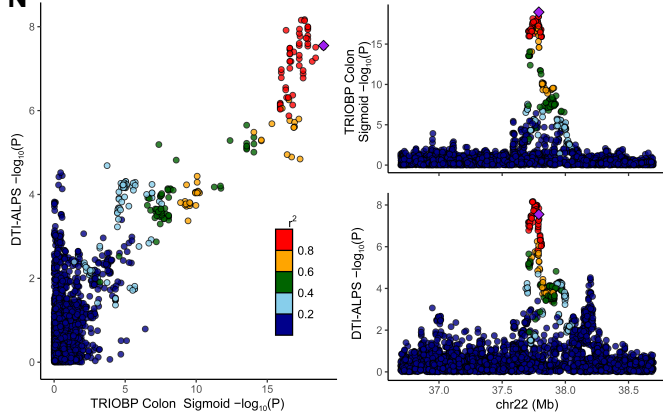**O**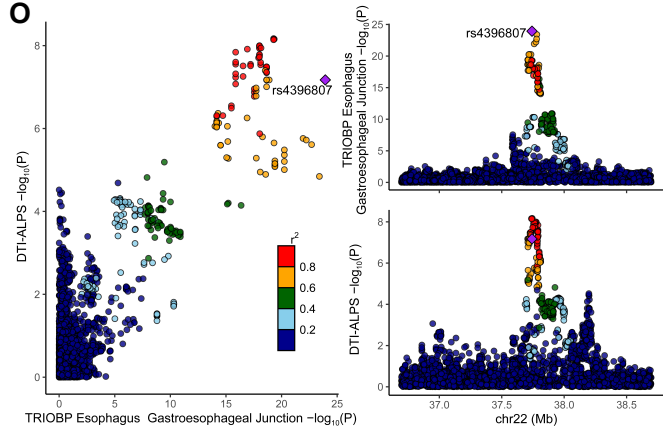**P**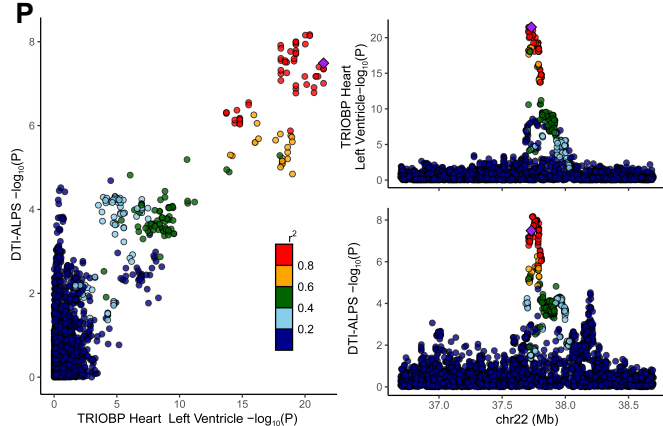

Supplement: Supplementary file 4 — Supplementary Material 4. [file 13041_2025_1258_MOESM4_ESM.pdf]

**Q**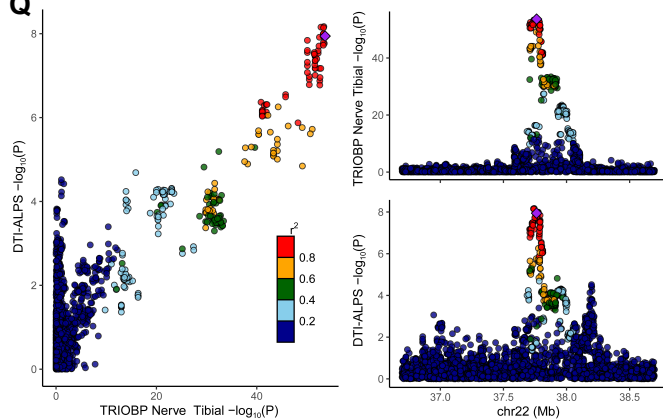**R**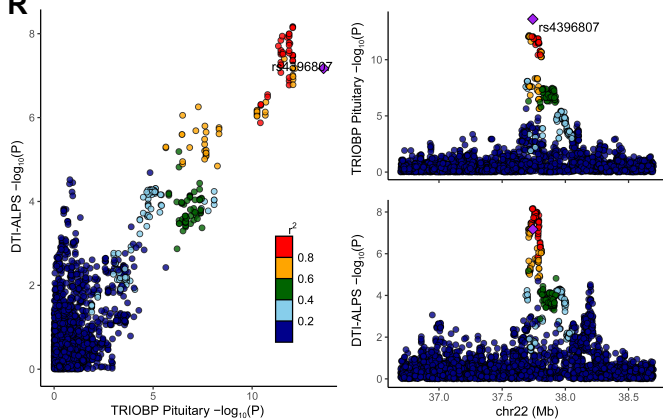**S**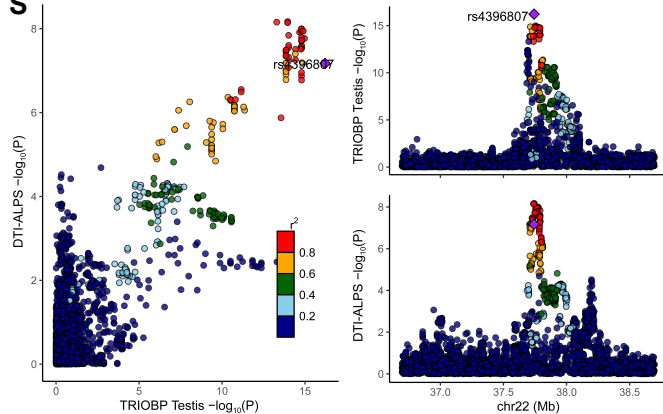**T**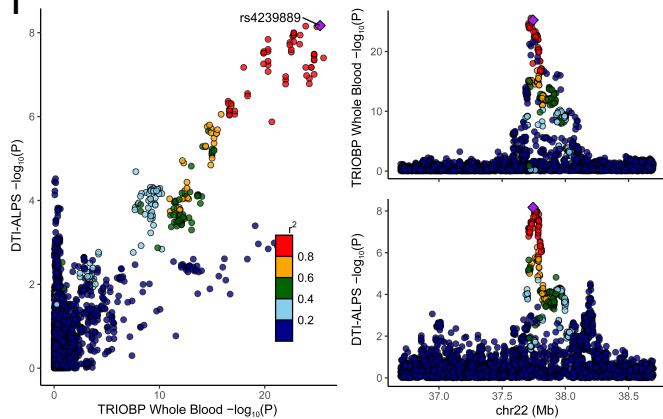

Supplement: Supplementary file 5 — Supplementary Material 5. [file 13041_2025_1258_MOESM5_ESM.pdf]

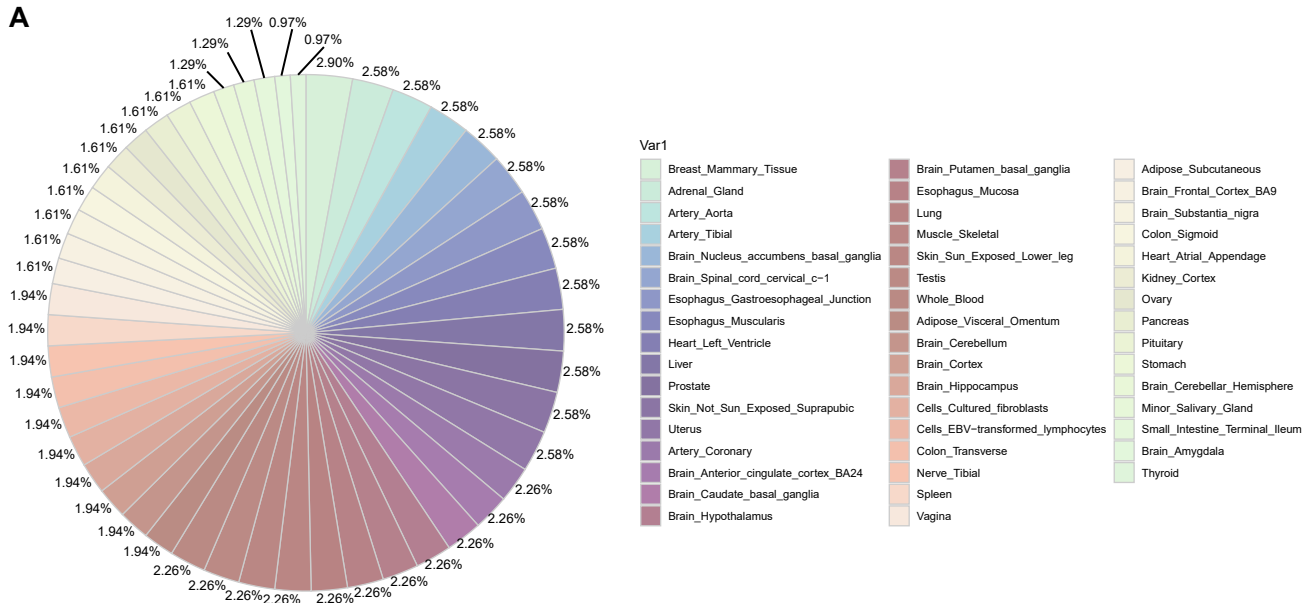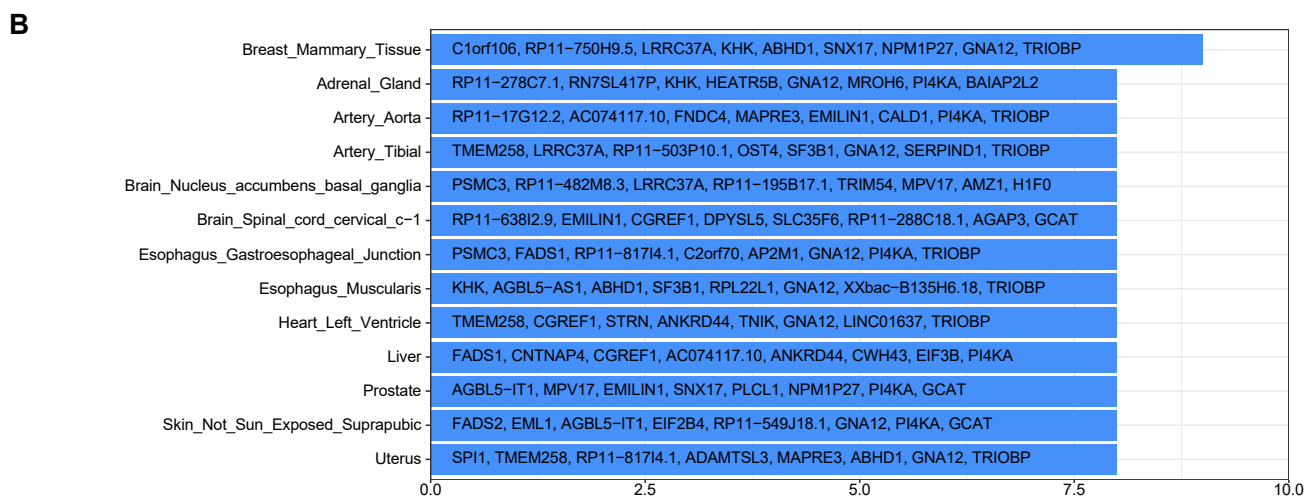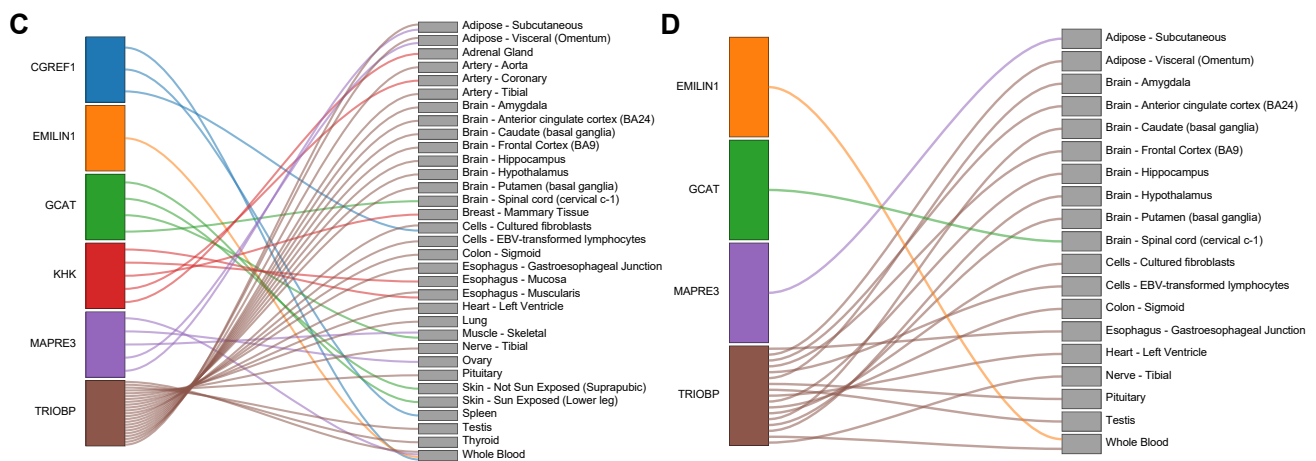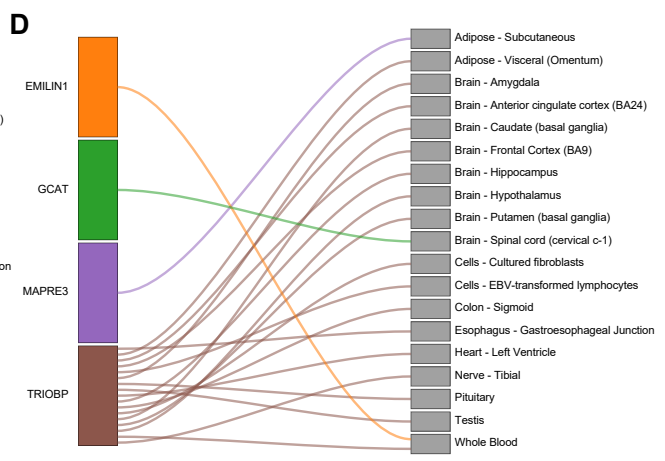

Supplement: Supplementary file 6 — Supplementary Material 6. [file 13041_2025_1258_MOESM6_ESM.pdf]
