# Supplementary material for: A cross-tissue transcriptome-wide association study identifies novel candidate genes associated with brain glymphatic system function
Source: Mol Brain. 2025 Dec 5;18:90. doi: 10.1186/s13041-025-01258-y (PMC12681178; doi:10.1186/s13041-025-01258-y)
Supplement: Supplementary file 8 — Supplementary Material 8. [file 13041_2025_1258_MOESM8_ESM.docx]

**Supplementary Figure Legend**

**Figure S1:** The results of Coloc analysis between candidate genes and DTI-ALPS Colocation of eQTL and GWAS associations for EMILN1 (A), GCAT (B), MAPRE3 (C) and TRIOBP (D-T). A scatterplot highlights the convergence of GWAS and eQTL associations for these genes.

**Figure S2.** The distribution of tissues corresponding to the previously identified single-tissue positive results. (A) Pie chart showing the distribution of tissue specificity identified by FUSION. (B) Bar chart displaying the gene names in the top 13 tissues with the highest number of genes identified by FUSION. (C) Sankey diagram illustrating the tissue distribution of positive genes identified by MR. (D) Sankey diagram illustrating the tissue distribution of positive genes identified by Coloc.
